# Learning curve in periacetabular osteotomy for developmental dysplasia of the hip: a systematic review and meta-analysis

**DOI:** 10.1302/2633-1462.74.BJO-2025-0371.R1

**Published:** 2026-04-22

**Authors:** Nikolai Ramadanov, Maximilian Heinz, Maximilian Voss, Robert Hable, Roland Becker, Sufian S. Ahmad

**Affiliations:** 1 Center of Orthopaedics and Traumatology, Brandenburg Medical School, University Hospital Brandenburg an der Havel, Brandenburg, Germany; 2 Faculty of Health Science Brandenburg, Brandenburg Medical School Theodor Fontane, Brandenburg an der Havel, Germany; 3 Faculty of Applied Computer Science, Deggendorf Institute of Technology, Deggendorf, Germany; 4 Department of Orthopaedic Surgery, Hannover Medical School, Hannover, Germany

**Keywords:** Periacetabular osteotomy, Learning curve, Surgeon experience, Developmental dysplasia of the hip, Operating time, Complications, Radiological correction, Hip preservation surgery, Meta-analysis, Developmental dysplasia of the hip (DDH), curve in periacetabular osteotomy, hips, blood loss, total hip arthroplasty (THA), hip preservation surgery, osteotomies, prospective studies, morbidity, periacetabular osteotomy

## Abstract

**Aims:**

Periacetabular osteotomy (PAO) is technically demanding with an assumed steep learning curve. This systematic review and meta-analysis evaluated how surgeon experience influences operative efficiency, perioperative morbidity, radiological correction, and conversion to total hip arthroplasty (THA) in developmental dysplasia of the hip (DDH).

**Methods:**

A systematic search of five databases identified studies comparing early with late PAO experience phases. Random-effects meta-analyses (Sidik-Jonkman with Hartung-Knapp adjustment) were performed for continuous (mean difference (MD)) and binary (odds ratios (ORs)) outcomes. Risk of bias was assessed with Risk Of Bias In Non-randomized Studies of Interventions (ROBINS-I), and certainty of evidence with Grading of Recommendations Assessment, Development, and Evaluation (GRADE).

**Results:**

In all, seven studies (499 patients, 556 hips) were included. Late-phase PAOs had significantly shorter operative times (MD –74.58 minutes, 95% CI –136.52 to –12.65). No significant differences were found for blood loss, complications, THA conversion, or radiological correction. Heterogeneity was high for operating time and blood loss, and most studies showed moderate risk of bias.

**Conclusion:**

Surgeon experience substantially improves operative efficiency in PAO, while complications, blood loss, radiological accuracy, and early THA conversion appear largely unaffected, likely reflecting structured supervision and high-volume training environments. The lack of significant differences in complications and radiological correction suggests that structured mentorship and centralized hip preservation programmes may mitigate early-phase risk. Standardized, prospective studies are needed to define proficiency thresholds and optimize training in hip preservation surgery.

Cite this article: *Bone Jt Open* 2026;7(4):574–583.

## Introduction

Developmental dysplasia of the hip (DDH) is a major cause of early osteoarthritis and functional limitation in young adults. Correcting acetabular malorientation before the onset of advanced degenerative changes is crucial for joint preservation. The Bernese periacetabular osteotomy (PAO), first described by Ganz et al,^1^ has become the preferred reconstructive procedure for symptomatic DDH with maintained joint congruency. Long-term follow-up studies demonstrate high patient satisfaction and durable survivorship exceeding 80% at 15 to 20 years.^2,3^

However, PAO is one of the most technically demanding procedures in hip preservation surgery. It requires precise osteotomies and controlled reorientation of the acetabular fragment under limited visualization, with a narrow margin for error. The complexity of the procedure naturally implies a steep learning curve, where surgical experience directly affects accuracy, complications, and efficiency.

Learning curves are well established in other orthopaedic domains. In hip arthroscopy, for instance, operating time and complication rates decline significantly after approximately 30 procedures, with further gains in efficiency and patient-reported outcomes beyond 100 cases.^4,5^ Meta-analyses in total hip arthroplasty (THA) have likewise shown that low surgeon or hospital volume is independently associated with higher dislocation, revision, and mortality rates.^6^ Comparable findings have been reported in pelvic and acetabular fracture fixation, where improved radiological reduction and lower complication rates are achieved only after considerable case exposure.^7,8^

Despite this consistent evidence linking surgical experience to improved outcomes across orthopaedic sub-specialties, a quantitative synthesis of the learning curve specific to PAO in DDH has been lacking. Understanding how surgeon experience influences operating time, blood loss, complication rates, and functional outcomes is essential to guide training, case supervision, and institutional volume requirements.

Therefore, the present systematic review and meta-analysis aims to evaluate the learning curve in periacetabular osteotomy for developmental dysplasia of the hip by pooling available data comparing early with late experience phases. This work intends to define the magnitude of improvement, identify the primary outcome domains affected by experience, and provide an evidence-based framework for training and quality assurance in PAO surgery.

## Methods

### Protocol and registration

This systematic review and meta-analysis was conducted in accordance with the PRISMA guidelines.^9^ The review protocol was registered prospectively in PROSPERO (CRD420251181503). The completed PRISMA checklist is provided in the Supplementary Material.

### Eligibility criteria

Studies were eligible for inclusion if they met all of the following criteria: 1) Patients with DDH or borderline dysplasia undergoing Bernese PAO; 2) PAO performed by surgeons with clearly distinguishable phases of operative experience (early vs late cases, defined by chronological order or cumulative sum (CUSUM) analysis); 3) Reporting at least one of the following: operating time, intraoperative blood loss, complication rate, conversion to THA, or radiological correction; 4) Prospective or retrospective cohort studies, case series, or registry-based analyses; and 5) English-language publications in peer-reviewed journals. Exclusion criteria comprised review articles, single case reports, technical notes, animal or cadaveric studies, and studies that did not distinguish surgeon experience phases.

### Search strategy

A comprehensive electronic literature search was conducted in PubMed/MEDLINE, Embase, Scopus, Web of Science, and Cochrane CENTRAL from database inception up to 31 December 2025. The search strategy combined controlled vocabulary and free-text terms related to PAO and learning-curve analysis. Database-specific search strategies for PubMed/MEDLINE, Embase, Scopus, Web of Science, and CENTRAL were developed according to the syntax and indexing of each database (Supplementary Material). Reference lists of all included studies and relevant reviews were screened manually for additional eligible records. Searches were conducted by two independent reviewers (NR, MV), with discrepancies resolved by consensus or third-party adjudication.

### Study selection

All retrieved records were de-duplicated manually and screened systematically. Two reviewers (NR, MV) independently assessed titles and abstracts, followed by full-text evaluation of all potentially eligible studies. Any disagreements were resolved through discussion, and unresolved conflicts were adjudicated by a senior reviewer.

### Data extraction

A standardized Excel (Microsoft, USA) extraction sheet was developed and piloted before data collection. Extracted items included study characteristics (author, year, country, design), sample size, patient demographic details, surgeon-experience definition, learning-curve methodology (chronological vs CUSUM), and all reported outcomes for early compared with late experience phases. All data were extracted directly from the published text, tables, and numerical results. Early and late experience phases were not redefined in the present study but they were adopted as reported in the primary studies. Corresponding authors were contacted in cases of missing or unclear information. A raw data extraction sheet is provided in the Supplementary Material.

### Risk of bias assessment

Two reviewers (MV, MH) independently assessed risk of bias using the Risk Of Bias In Non-randomized Studies of Interventions (ROBINS-I) tool^10^ for non-randomized studies. Disagreements were resolved through consensus. Overall certainty of evidence across outcomes was evaluated using the Grading of Recommendations Assessment, Development, and Evaluation (GRADE)^11^ framework.

### Data synthesis and statistical analysis

A frequentist meta-analysis was performed when at least three studies reported comparable outcomes. Pooled effect sizes were calculated using a random-effects model with the Sidik-Jonkman estimator^12^ and Hartung-Knapp adjustment.^13^ For continuous outcomes (operating time, intraoperative blood loss), mean differences (MD) with 95% CIs were used. Dichotomous outcomes (complications, THA conversion, radiological correction) were synthesized using odds ratios (ORs). Between-study heterogeneity was quantified using I², τ², and the Q statistic. Planned sub-group analyses examined effect differences between early and late experience phases, study design, and surgeon training status. Publication bias was assessed by visual inspection of funnel plots.^14^ Statistical significance was set at p < 0.05.

## Results

### Systematic literature search

**Fig. 1:**
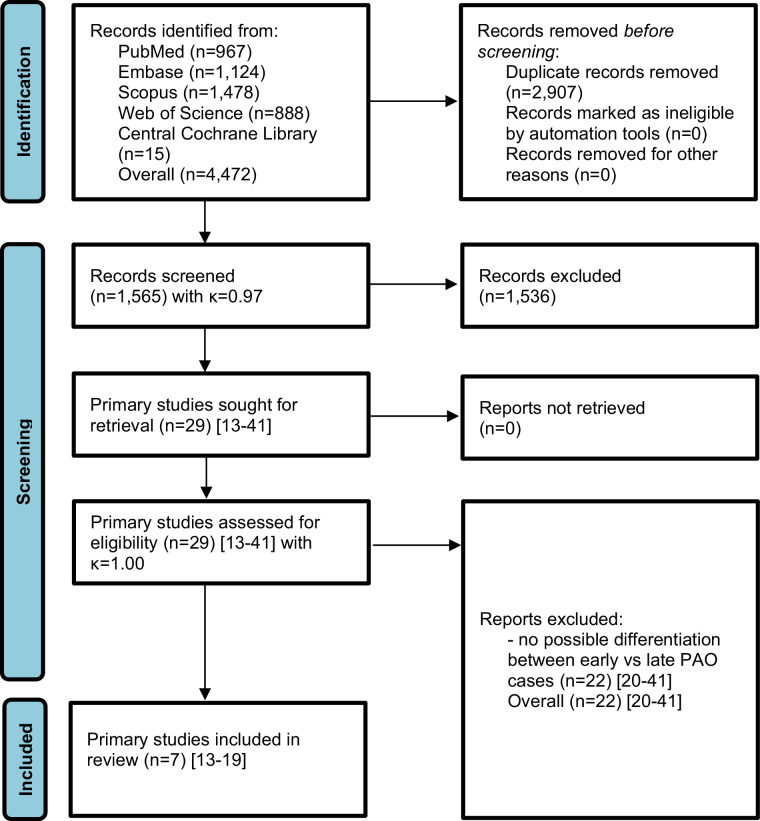
PRISMA flow diagram of study selection. Flow diagram illustrating the identification, screening, eligibility assessment, and inclusion of studies. A total of 4,472 records were identified, 2,907 duplicates were removed, and 29 full-text articles^15-43^ were assessed for eligibility. Ultimately, seven primary studies^15-21^ met all criteria and were included in the quantitative synthesis.

The literature search of PubMed (n = 967), Embase (n = 1,124), Scopus (n = 1,478), Web of Science (n = 888), and Central Cochrane CENTRAL Library (n = 15) yielded overall 4,472 records for title and abstract screning with high inter-reviewer agreement (κ = 0.97) after removal of 2,907 duplicates. A total of 29 primary studies^15-43^ were assessed for eligibility with full inter-reviewer agreement (κ = 1.0), whereas 22 primary studies were excluded because they did not report early compared with late case series of PAO.^22-43^ A detailed list of full-text studies excluded after eligibility assessment, including reasons for exclusion, is provided in the Supplementary Material. The systematic literature search identified seven primary studies^15-21^ with a total of 499 patients (556 hips) that met the eligibility criteria for inclusion in the meta-analysis (Figure 1).

### Patient characteristics

The seven included primary studies comprised 499 patients undergoing 556 PAOs, with 202 early and 354 late cases. The weighted mean age across studies was 25.2 years (SD 6.8; 10 to 53), and 82.6% of all patients were female. The mean BMI was 25.2 kg/m² (SD 5.0; 17 to 38). All procedures were Bernese PAOs^15-21^ performed for DDH or borderline DDH. Reported preoperative outcome measures included the Merle d’Aubigné-Postel score with 12.4 points,^15^ modified Harris Hip Score (HHS) with 61.0 points,^16^ and HHS with 54 points.^21^ The mean follow-up across studies was 42.9 months (16 to 300). Further details on individual study characteristics are summarized in Table I.

**Table I.** Characteristics of included primary studies. Summary of baseline study characteristics for all included primary studies,^15-21^ including study design, country, sample size, patient demographic details, definition of early compared with late experience phases, learning-curve methodology, follow-up duration, and preoperative outcome measures.

First author	Origin	Journal (ISSN)	Study design	Level of evidence	Patients, n	Hips, n	Early cases, n	Late cases, N	Mean age, yrs (SD; range)	Female sex, n (%)	Mean BMI, kg/m² (SD; range)	Indication	Preoperative outcome parameters, points	PAO type	Follow-up period, mths
Burke et al 2011^15^	Ireland — Cappagh National Orthopaedic Hospital & Royal College of Surgeons in Ireland, Dublin	Acta Orthopaedica Belgica (0001 to 6462)	Retrospective cohort study	IV	79	85	42	43	22.9 (14 to 41)	72 (91.1)	N/A	DDH	Merle d'Aubigné-Postel hip score. 12.4	Bernese	59 (16 to 96)
Chou et al 2019^16^	Australia — University of Adelaide & Royal Adelaide Hospital, South Australia	Clinical Orthopaedics and Related Research (0009 to 921 X)	Retrospective cohort study	IV	72	85	26	59	26 (14 to 45)	54 (75.0)	24.7 (6.4; 18 to 37)	DDH	mHHS: 61 (13)	Bernese	60 (24 to 300)
Grammatopoulos et al 2016^17^	UK — Nuffield Orthopaedic Centre, University of Oxford	Clinical Orthopaedics and Related Research (0009 to 921 X)	Retrospective case series	III	57	68	20	48	25 (7; 15 to 41)	49 (86.0)	25.1 (4.8; 18 to 34)	DDH, BDDH	N/A	Bernese	96 (24 to 216)
Haertlé et al 2024^18^	Germany — Hannover Medical School (MHH), Hannover	Bone & Joint Journal (2049 to 4408)	Retrospective cohort study	III	106	118	26	92	23.4 (7.5; 16 to 53)	90 (84.9)	24.9 (4.6; 17 to 38)	DDH, BDDH	N/A	Bernese	3
McKinley et al 2003^19^	USA — University of Iowa Hospital and Clinics, Iowa City, Iowa	Iowa Orthopaedic Journal (0149 to 1728)	Retrospective case series	IV	31	36	18	18	33 (7.9; 15 to 47)	24 (77.4)	N/A	DDH	N/A	Bernese	24
Novais et al 2017^20^	USA — Children’s Hospital Colorado & Children’s Hospital of Philadelphia (in collaboration with Boston Children’s Hospital, Harvard University)	Clinical Orthopaedics and Related Research (0009 to 921 X)	Retrospective cohort study	III	81	81	40	41	18 (5; 10 to 36)	68 (84.0)	23 (4)	DDH	N/A	Bernese	12
Peters et al 2006^21^	Department of Orthopaedics, University of Utah, Salt Lake City, Utah, USA	The Journal of Bone and Joint Surgery (1535 to 1386)	Retrospective case series	IV	73	83	30	53	28 (15 to 47)	55 (75.3)	28.5 (17.1 to 33.9)	DDH	HHS: 54 (range 20 to 81)	Bernese	46 (30 to 88)
Overall descriptive summary of included studies	N/A	N/A	N/A	N/A	499	556	202	354	25.2 (6.8; 10 to 53)	412 (82.6)	25.2 (5.0; 17 to 38)	N/A	N/A	N/A	42.9 (16 to 300)

BDDH, borderline developmental dysplasia of the hip; DDH, developmental dysplasia of the hip; HHS, Harris Hip Score; mHHS, modified Harris Hip Score; N/A, not available; PAO, periacetabular osteotomy .

### Quality assessment 

Of the seven primary studies^15-21^ included, four were rated with a moderate risk of bias,^15-17,21^ two were rated with a low risk of bias^17,20^ (Table II), and one was rated with a serious risk of bias.^19^ Funnel plots for all outcomes (Supplementary Material) showed generally symmetrical distributions without obvious small-study effects; however, due to the limited number of studies per outcome (k ≤ 4), visual assessment remains inconclusive.

**Table II.** Risk of bias assessment using the ROBINS-I tool. Risk-of-bias ratings for all included non-randomized studies across seven ROBINS-I domains: confounding, selection of participants, classification of interventions, deviations from intended interventions, missing data, outcome measurement, and selection of reported results. Overall risk-of-bias judgements for each study are presented.

Study	D1 Confounding	D2 Selection	D3 Classification	D4 Deviations	D5 Missing data	D6 Outcome measurement	D7 Reporting bias	Overall
Burke et al 2011^15^	Moderate	Moderate	Moderate	Moderate	Moderate	Moderate	Moderate	Moderate
Chou et al 2019^16^	Moderate	Moderate	Moderate	Low	Low	Moderate	Moderate	Moderate
Grammatopoulos et al 2016^17^	Moderate	Low	Moderate	Low	Moderate	Moderate	Moderate	Moderate
Haertlé et al 2024^18^	Low	Low	Low	Low	Low	Low	Low	Low
McKinley et al 2003^19^	Serious	Moderate	Moderate	Moderate	Moderate	Moderate	Moderate	Serious
Novais et al 2017^20^	Low	Low	Low	Low	Low	Low	Low	Low
Peters et al 2006^21^	Moderate	Moderate	Moderate	Low	Low	Moderate	Moderate	Moderate

ROBINS-I, Risk Of Bias In Non-randomized Studies – of Interventions.

### Meta-analysis

**Fig. 2:**
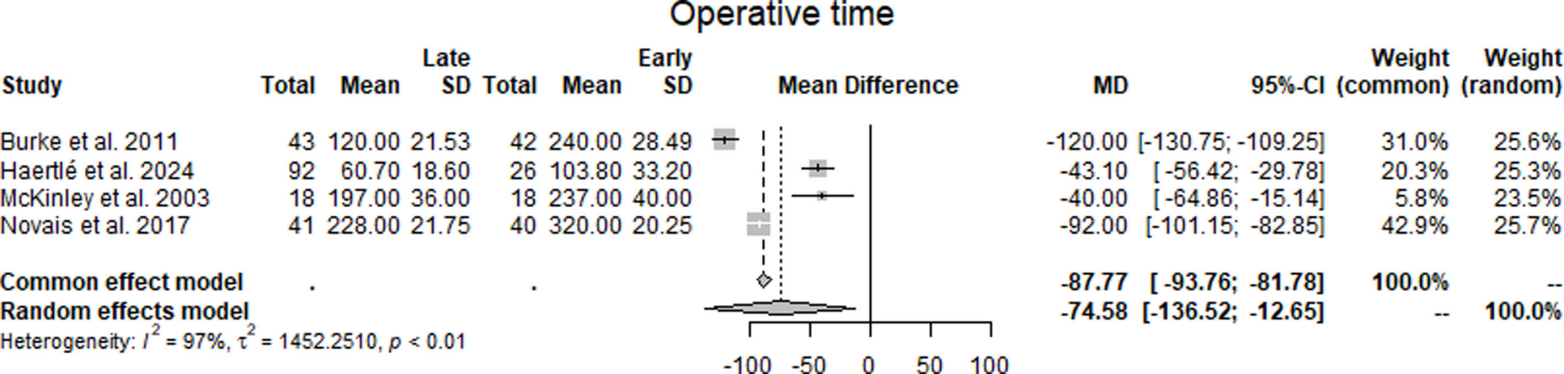
Forest plot of operating time (min) comparing early with late periacetabular osteotomy cases, and showing mean differences (MDs) in operating time across four studies.^15,18-20^ Negative values represent shorter operative times in late cases. The pooled random-effects estimate was –74.58 minutes (95% CI –136.52 to –12.65), with high heterogeneity (I² = 97%).

Operating time: Across four studies^15,18-20^ reporting operating time, the mean differences between late and early cases were –120.0 minutes (Burke et al),^15^ –43.1 minutes (Haertlé et al),^18^ –40.0 minutes (McKinley et al),^19^ and –92.0 minutes (Novais et al).^20^ The pooled estimate under the random-effects model was –74.58 minutes (95% CI –136.52 to –12.65), representing a statistically significant difference. Statistical heterogeneity was high (I² = 97%, τ² = 1452.25, p < 0.01) (Figure 2, Table III).

**Table III.** Summary of pooled outcomes from the meta-analysis.

Parameter	Primary studies, n	Hips, n	Treatment effect	95% CI	p-value	I^2^	τ^2^	Egger bias	Egger p-value
Operating time	4	320	-74.58	-136.52 to -12.65	0.031*	0.97	1452.25	9.42	0.399
Blood loss	2	121	-753.54	-5,318.41 to 3,811.34	0.283	0.91	235,166.98	N/A	
Complications	5	403	0.45	0.1 to 2.06	0.218	0.62	0.75	-1.39	0.582
THA conversion	4	556	1.17	0.06 to 23.84	0.879	0.44	1.34	0.29	0.954
Radiological outcome	4	390	3.01	0.75 to 11.99	0.085	0.00	0.06	-2.95	0.036*

Summary of pooled effect sizes for all evaluated outcomes, including operating time (mean difference), intraoperative blood loss (mean difference), complication rates (odds ratio; OR), conversion to total hip arthroplasty (OR), and radiological correction (OR). Corresponding 95% CIs, heterogeneity statistics (I², τ²), and number of contributing studies per outcome are shown.

* Significant at p < 0.05.

N/A, not available; THA, total hip arthroplasty.

**Fig. 3:**
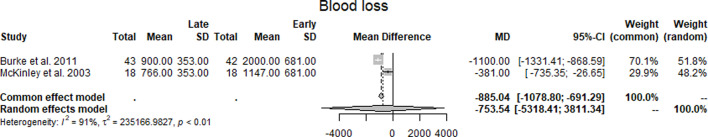
Forest plot of intraoperative blood loss (ml) comparing early with late periacetabular osteotomy cases, presenting mean differences (MDs) in intraoperative blood loss across two studies.^15,19^ Negative values indicate lower blood loss in late cases. The pooled random-effects estimate was –753.54 ml (95% CI –5,318.41 to 3,811.34), with high heterogeneity (I² = 91%). MD, mean difference.

Blood loss: Across two studies^15,19^ reporting intraoperative blood loss, the mean differences between late and early cases were –1,100.0 ml (Burke et al)^15^ and –381.0 ml (McKinley et al).^19^ The pooled estimate under the random-effects model was –753.54 ml (95% CI –5,318.41 to 3,811.34), which was not statistically significant. Statistical heterogeneity was high (I² = 91%, τ² = 235,166.98, p < 0.01) (Figure 3, Table III).

**Fig. 4:**
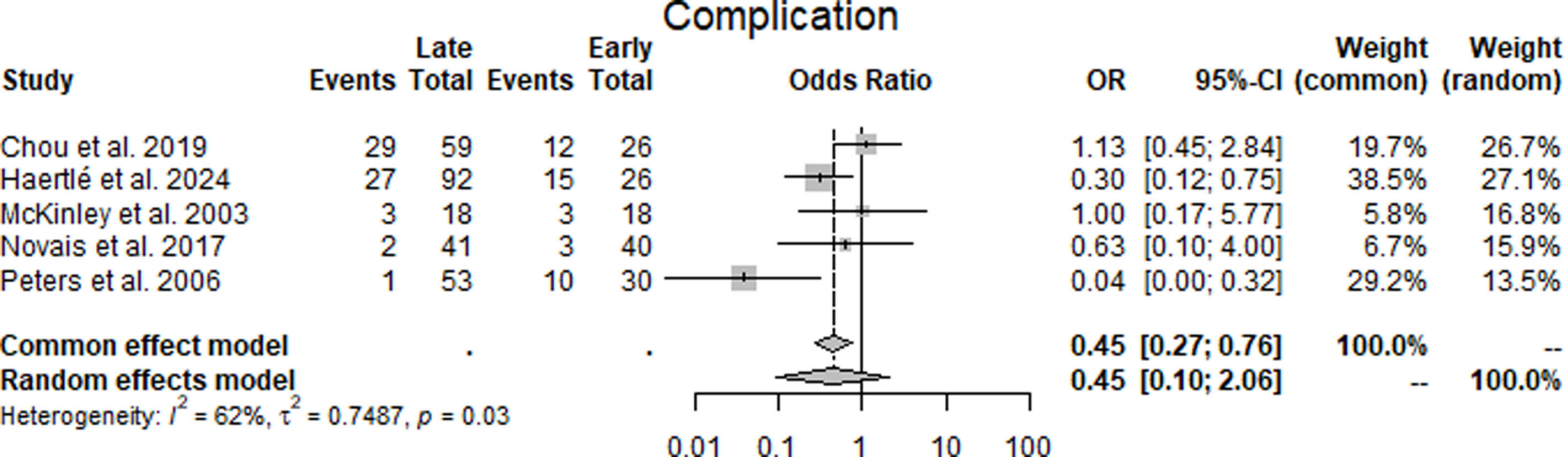
Forest plot of complication rates comparing early with late periacetabular osteotomy cases, displaying odds ratios (ORs) for complications across five studies.^16,18-21^ An OR of < 1 indicates fewer complications in late cases. The pooled random-effects estimate was OR 0.45 (95% CI 0.10 to 2.06), with moderate heterogeneity (I² = 62%).

Complications: Across five studies^16,18-21^ reporting complications, the odds ratios comparing late with early cases were 1.13 (Chou et al),^16^ 0.30 (Haertlé et al),^18^ 1.00 (McKinley et al),^19^ 0.63 (Novais et al),^20^ and 0.04 (Peters et al).^21^ The pooled estimate under the random-effects model was 0.45 (95% CI 0.10 to 2.06), which was not statistically significant. Statistical heterogeneity was moderate (I² = 62%, τ² = 0.7487, p = 0.03) (Figure 4, Table III).

**Fig. 5:**
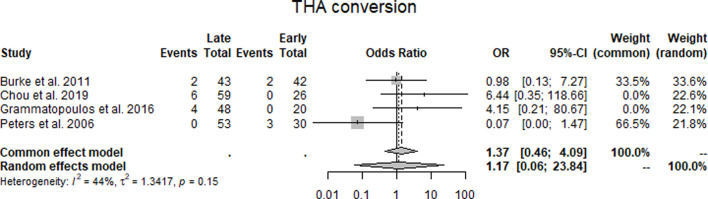
Forest plot of conversion to total hip arthroplasty (THA) comparing early with late periacetabular osteotomy cases. Forest plot reporting odds ratios (ORs) for conversion to THA across four studies.^15-17,21^ The pooled random-effects estimate was OR 1.17 (95% CI 0.06 to 23.84), with moderate heterogeneity (I² = 44%).

THA conversion: Across four studies^15-17,21^ reporting conversion to THA, the odds ratios comparing late with early cases were 0.98 (Burke et al),^15^ 6.64 (Chou et al),^16^ 4.15 (Grammatopoulos et al),^17^ and 0.07 (Peters et al).^21^ The pooled estimate under the random-effects model was 1.17 (95% CI 0.06 to 23.84), which was not statistically significant. Statistical heterogeneity was moderate (I² = 44%, τ² = 1.3417, p = 0.15) (Figure 5, Table III).

**Fig. 6:**
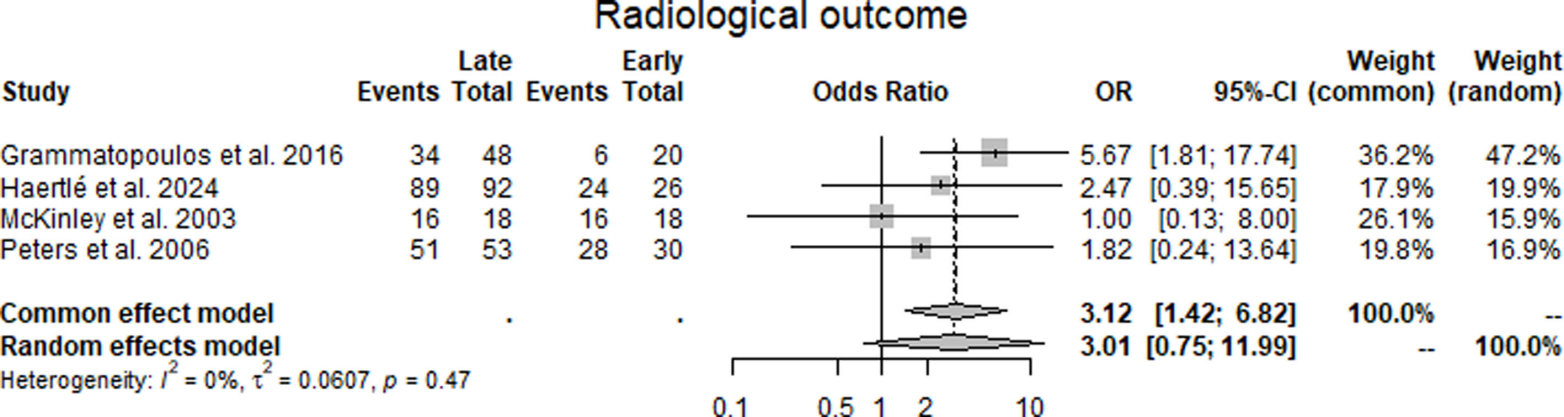
Forest plot of radiological correction comparing early with late periacetabular osteotomy cases, and presenting odds ratios (ORs) for achieving radiological correction across four studies.^17-19,21^ The pooled random-effects estimate was OR 3.01 (95% CI 0.75 to 11.99), with low heterogeneity (I² = 0%).

Radiological outcome: Across four studies^17-19,21^ reporting radiological outcomes, the odds ratios comparing late with early cases were 5.67 (Grammatopoulos et al),^17^ 2.47 (Haertlé et al),^18^ 1.00 (McKinley et al),^19^ and 1.82 (Peters et al).^21^ The pooled estimate under the random-effects model was 3.01 (95% CI 0.75 to 11.99), which was not statistically significant. Statistical heterogeneity was low (I² = 0%, τ² = 0.0607, p = 0.47) (Figure 6, Table III).

## Discussion

This systematic review and meta-analysis synthesized the available evidence on the influence of surgeon experience on outcomes following periacetabular osteotomy for developmental dysplasia of the hip. Seven primary studies comprising 499 patients and 556 hips were included. Across four studies, operating time was significantly reduced in late compared with early cases (MD –74.6 minutes, 95% CI –136.5 to –12.7). No statistically significant differences were observed for intraoperative blood loss, complications, conversion to THA, or radiological correction. Heterogeneity varied across outcomes, ranging from low (radiological outcomes) to high (operating time, blood loss). Overall, the included studies demonstrated predominantly moderate risk of bias, and the certainty of evidence remained limited by the small number of available studies and variability in reporting.

### Interpretation of findings

The findings indicate that surgical efficiency improves substantially with increasing experience, consistent with the expected trajectory of a high-complexity pelvic realignment procedure. The marked reduction in operating time reflects accumulated familiarity with osteotomy planes, acetabular fragment mobilization, intraoperative imaging, and periacetabular anatomy. Although blood loss tended to be lower in late cases, the pooled estimate did not reach statistical significance, likely due to wide confidence intervals and limited sample size.

The very high heterogeneity observed for operating time and blood loss is likely multifactorial. Contributing factors include substantial variation in the definition of early compared with late experience (chronological cutoffs vs CUSUM analyses), heterogeneous patient populations (adolescent and adult cohorts), and inconsistently reported levels of supervision and institutional experience. Additional variability in surgical technique and perioperative blood management protocols further limits comparability across studies.

For safety-related outcomes (complications, reoperations leading to THA, and radiological accuracy), no statistically significant differences were detected between early and late series. This may imply that early-phase PAOs performed in high-volume centres or by surgeons under supervision achieve clinically acceptable outcomes despite longer operative duration. Alternatively, the absence of significant differences may simply reflect insufficient statistical power, given that most outcomes were reported in only two to five studies.

Radiological correction did not differ significantly between early and late phases. This suggests that although technical execution becomes more efficient, the capacity to obtain adequate acetabular reorientation may be achievable early, particularly under specialized training structures. However, the wide variability in how ‘adequate correction’ was defined across studies limits definitive conclusions.

Overall, the pattern across outcomes is consistent with a procedural learning curve that primarily manifests through improved operative efficiency, while clinical and radiological outcomes remain broadly comparable across experience levels.

### Comparison with the literature

The results align with learning-curve phenomena documented across orthopaedic sub-specialties. In hip arthroscopy, cumulative operative efficiency improves markedly with experience, with reductions in surgical and traction time and a parallel decline in complications and revisions after approximately 30 to 110 cases.^4,5^ While PAO differs fundamentally from arthroscopy in complexity and anatomical exposure, both procedures demonstrate early improvements in technical efficiency followed by slower gains in precision-based metrics.

In THA, surgeon and hospital volume correlate with dislocation, revision, and mortality risk,^6^ underscoring the broader principle that procedural repetition enhances both efficiency and clinical outcomes. Similar patterns are reported in pelvic and acetabular trauma surgery, where improved reduction quality and reduced complications occur only after considerable case exposure.^7,8^ The present findings fit within this paradigm, although the effect size for PAO appears to be confined primarily to operating time.

PAO outcomes are influenced not only by surgical experience, but also by patient-specific anatomical factors. Pelvic tilt remains largely unchanged after PAO, suggesting biomechanical stability that may blunt learning-curve effects.^44^ In borderline dysplasia, morphology-driven predictors outweigh technical factors, which may explain the limited impact of surgeon experience on complications and radiological outcomes.^45,46^ Beyond surgeon experience, technical details of PAO execution may exert a substantial influence on procedure-related risks. A recent 3D CT simulation study^47^ demonstrated that the position of the pubic cut is a major determinant of pubic root displacement during PAO, with a ten-fold reduction in complete displacement when a lateral cut was used. These findings highlight that specific technical decisions, independent of surgical experience, may contribute to postoperative complications such as delayed union or anterior hip syndrome.

Taken together, the existing literature suggests that PAO learning-curve effects manifest most clearly in procedural efficiency, while structural and patient-level determinants have a predominant influence on clinical and radiological outcomes. This contextual understanding helps explain why our analysis identified substantial improvements in operating time but no consistent differences in safety or correction metrics between early and late experience phases.

### Clinical implications

These findings have several implications for surgical training, case allocation, and institutional practice. First, since operating time improves substantially with experience, early-phase PAOs should be planned with appropriate scheduling buffers and close supervision by experienced surgeons. Second, the lack of significant differences in complications and radiological correction suggests that structured mentorship and centralized hip preservation programmes may mitigate early-phase risk. Third, the data support the recommendation that PAO training should occur in high-volume centres where trainees experience adequate case exposure to progress along the steep portion of the learning curve. Finally, outcome monitoring frameworks should consider operative efficiency as a primary marker of learning progression, while recognizing that radiological accuracy and patient outcomes appear less sensitive to early experience under supervised conditions.

Strengths of this study include the strict application of PRISMA methodology, a pre-registered PROSPERO protocol, and the use of random-effects modelling appropriate for small samples and between-study variability. Independent dual reviewer assessment minimized selection and extraction bias, and risk of bias was evaluated using established tools (ROBINS-I, GRADE).

Limitations include the small number of studies per outcome and substantial heterogeneity for several analyses. Definitions of early compared with late experience varied widely (chronological cutoffs vs CUSUM analyses), and reporting of key variables, particularly postoperative patient-reported outcome measures (PROMs), was inconsistent. Most included studies were retrospective and subject to selection and reporting bias. Radiological correction is a major limitation of this analysis, as definitions varied substantially across studies, limiting the validity and interpretability of the pooled estimate, which should therefore be interpreted with caution. The moderate to serious risk of bias identified by ROBINS-I limits causal interpretation, as residual confounding, non-random case selection, and non-standardized classification of experience phases may distort observed learning-curve effects. Finally, statistical power was limited, and some non-significant pooled estimates may reflect insufficient sample size rather than absence of true differences.

### Future directions

Future research should prioritize prospective multicentre registries with standardized definitions of early and late training phases, consistent radiological and clinical outcome reporting, and inclusion of surgeon-level covariates such as fellowship training, annual volume, and supervision structure. Integration of cumulative summation (CUSUM) and risk-adjusted models may provide more precise benchmarks for proficiency thresholds in PAO. Long-term PROMs and survivorship analyses across the learning curve remain largely unexplored and should be addressed in future work.

In conclusion, this systematic review and meta-analysis demonstrates that operative efficiency in periacetabular osteotomy improves with increasing surgeon experience, with consistently shorter operating times observed in late cases. In contrast, differences in blood loss, complications, and radiological outcomes between early and late cases were not statistically significant. While heterogeneity and methodological variability should be considered, the findings suggest that learning-curve effects in PAO primarily manifest through improved operative efficiency.

**Take home message**

- This study demonstrates that increasing surgeon experience in periacetabular osteotomy (PAO) primarily improves operative efficiency, with substantially shorter operating times, while perioperative safety and radiological correction remain largely unaffected.

- These findings suggest that, within structured training environments and under appropriate supervision, PAO can be performed safely even during the early learning phase. This supports the centralization of hip preservation surgery and emphasizes the importance of standardized training pathways.

## Data Availability

All data generated or analyzed during this study are included in the published article and/or in the supplementary material.
